# Vertical stratification of *Culicoides* biting midges at a Florida big game preserve

**DOI:** 10.1186/s13071-018-3080-5

**Published:** 2018-09-10

**Authors:** Bethany L. McGregor, Alfred E. Runkel, Samantha M. Wisely, Nathan D. Burkett-Cadena

**Affiliations:** 1 Florida Medical Entomology Laboratory, University of Florida, 200 9th St. SE, Vero Beach, FL USA; 2 Department of Wildlife Ecology and Conservation, University of Florida, 110 Newins-Ziegler Hall, Gainesville, FL USA

**Keywords:** *Culicoides*, Canopy, Trap height, Vertical stratification, Hemorrhagic disease

## Abstract

**Background:**

Many important vector arthropods are known to stratify vertically in forest environments, a phenomenon which has important implications for vector-borne disease transmission and vector control. *Culicoides* Latreille biting midges (Diptera: Ceratopogonidae) have been documented using the forest canopy; however, studies of this phenomenon are lacking for many *Culicoides* species found in great abundance in the state of Florida, USA, some of which have been implicated as suspected vectors of hemorrhagic diseases of white-tailed deer. The present study aimed to determine whether common *Culicoides* species in Florida stratify vertically and to determine whether strata used by midges corresponded to host use.

**Methods:**

Trapping was conducted at a big game preserve in Gadsden County, FL, USA. Over two summer field seasons in 2016 and 2017, CDC miniature light traps were set at two levels, one set at 1.37 m, designated as the ground trap, and a nearby trap in the forest canopy set at 6 m during 2016 and 9 m during 2017. Species abundance, physiological status, and blood-meal sources were analyzed and compared between trap heights.

**Results:**

Species abundances for *C. haematopotus*, *C. stellifer* and *C. venustus* were not significantly different between trap heights during the 2016 season; however, canopy traps were found to have significantly higher abundance of *C. arboricola*, *C. biguttatus*, *C. debilipalpis*, *C. haematopotus*, *C. insignis* and *C. stellifer* than ground traps in 2017. Greater numbers of blood-engorged midges were collected in the canopy compared with ground traps during both study years, and 98.6% and 98.7% of blood meals from canopy collected midges were taken from ground-dwelling mammals in 2016 and 2017, respectively.

**Conclusions:**

*Culicoides* species in Florida, including species implicated as vectors of hemorrhagic disease viruses, are found in great abundance in the forest canopy. Many midges are feeding on host species that are known reservoirs of hemorrhagic disease and then moving into the forest canopy, which has implications for the calculation of vectorial capacity. These data contribute valuable ecological information on *Culicoides* species found in Florida and provide a framework for developing effective vector control strategies to target these species.

**Electronic supplementary material:**

The online version of this article (10.1186/s13071-018-3080-5) contains supplementary material, which is available to authorized users.

## Background

The distribution of vectors in the environment has a strong impact on interactions with host animals, the efficacy of vector control activities, and the transmission of vector-borne pathogens. Investigations into the vertical distribution of arthropods have found that taxa stratify by species, life stage, and physiological status [1], a phenomenon which has been demonstrated in many medically important dipteran families, including the Culicidae, Simuliidae, Ceratopogonidae, Muscidae and the subfamily Phlebotominae [2–4]. In addition, field studies from Connecticut, USA, found that West Nile virus minimum infection rates were significantly greater in two vector mosquito species in canopy-level traps, compared to traps at ground level [5]. Monitoring activities for the detection and prevention of arboviral outbreaks are most effective when optimized trapping protocols are employed. Despite this, many studies conducted on arboviral vectors focus trapping efforts within 1.5 m of the ground without investigating optimal trap heights for specific vector species, potentially missing insects of medical and veterinary importance that are present at higher vertical strata.

*Culicoides* Latreille biting midges (Diptera: Ceratopogonidae) are important vectors of pathogens of primarily veterinary importance, including epizootic hemorrhagic disease virus (EHDV), bluetongue virus (BTV), and African horse sickness virus (AHSV) [6]. They are also competent vectors of human pathogens in the New World, including Oropouche virus [7] and the filarial nematode *Mansonella ozzardi* [8]. The status of *Culicoides* as vectors of over 50 pathogens worldwide [6] necessitates developing optimized monitoring strategies not only in a horizontal plane, but also in vertical space.

Several studies have examined the use of vertical space by *Culicoides* species worldwide [9–11]. Height based differences in total abundance, physiological status, and species composition have been recorded in *Culicoides* [9]. In Africa, abundance and physiological status varied with height differences of less than 1 m [9]. The human pest, *Culicoides furens* Poey, was collected in great abundance in the canopy in South Carolina, USA, but collection heights depended on the habitat from which they were collected [11]. Thus, height at which *Culicoides* are trapped affects estimates of abundance, diversity and species composition.

While the precise biological rationale for *Culicoides* exploiting the forest canopy is not known, one hypothesis is that midges use the same vertical strata as their preferred host taxa [12]. By this reasoning, hematophagous insects that primarily parasitize large mammals would be collected in greater abundance near the ground [13] compared with those insects that prefer to feed on avian hosts, especially during host-seeking physiological stages - nulliparous and parous females [13, 14]. These associations have led to the proposal that host preferences can be inferred through the collection of hematophagous insects at different vertical strata [15]. Other research seemingly indicates that midges are simply feeding on whichever host is most abundant at their preferred vertical strata, and therefore the elevation of feeding determines host affiliation [11, 16].

Understanding the ecology of nuisance and vector species is an important step in developing management and maintenance plans. This information is very limited for many Florida *Culicoides* species, including species that have been implicated as vectors of hemorrhagic diseases such as EHDV and BTV. The present research aimed to investigate whether diverse *Culicoides* species are encountered in large numbers in tree canopies, whether physiological statuses of midges differ in those captured at ground versus canopy level, and whether blood-meal analysis could be used to assess the hypothesis that hematophagous *Culicoides* spp*.* are caught in highest abundance at the level of their preferred host class.

## Methods

### Field site

**Fig. 1:**
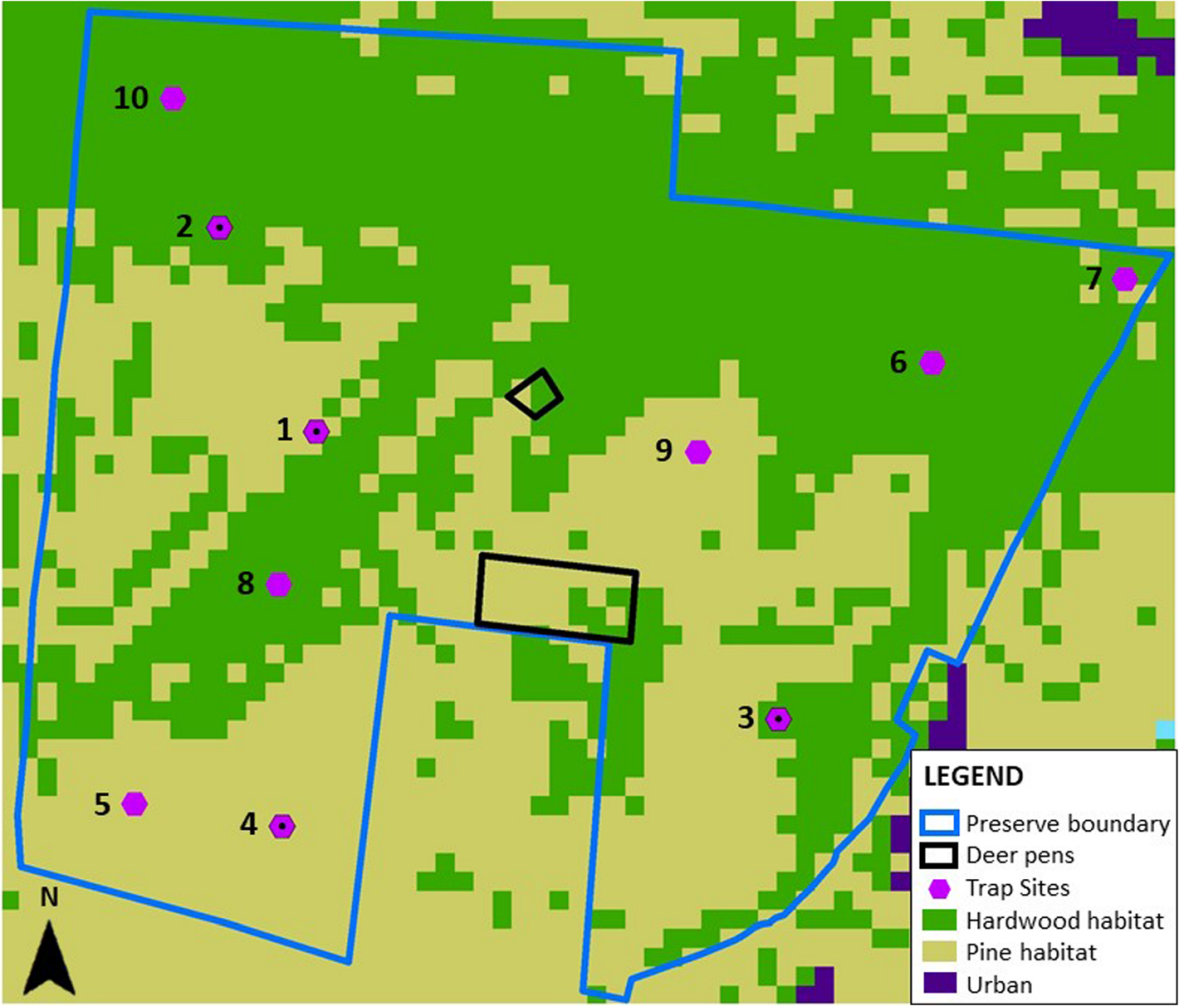
Map depicting property boundaries, habitat classes, and trap sites at a big game farm. Big game preserve located in Gadsden county, Florida. Each trap site (purple hexagons; sites with black dots were run during both 2016 and 2017, sites without black dots were only run during 2017) had two CDC miniature light traps present, one at ground level and one in a tree canopy. Funnel traps for passive collection of *Culicoides* moving up into and down from the canopy were also located at trap sites 2 and 3

Field experiments were conducted on a ~200-hectare big game preserve located in Gadsden County, FL. Dominant habitats included upland pine scrublands and bottomland hardwoods (Fig. 1). The property was mainly composed of a large open preserve where various species of big game (Cervidae and Bovidae) were free to roam. Cervid species present included axis deer (*Axis axis*), elk (*Cervus canadensis*), fallow deer (*Dama dama*), sika deer (*Cervus elaphus*), sika × elk hybrids (*Cervus elaphus* × *Cervus canadensis*) and white-tailed deer (*Odocoileus virginianus*). Bovid species included blackbuck antelope (*Antilope cervicapra*), bighorn sheep (*Ovis canadensis*), gemsbok (*Oryx gazella*), nilgai (*Boselaphus tragocamelus*), scimitar-horned oryx (*Oryx dammah*) and waterbuck (*Kobus ellipsiprymnus*).

### Experimental design

Trapping was conducted at four sites in 2016 and ten sites in 2017 (Fig. 1). At each trap site, Centers for Disease Control miniature light traps (Model 2836BQ, BioQuip Inc., Rancho Dominguez, USA) with LED black light arrays (Model 2790V390, BioQuip Inc., Rancho Dominguez, USA) were set up at two heights. No additional attractants were used for the traps. One trap was hung on a steel shepherd's hook with intake at 1.37 m and represented the “ground” trap during both study years. At each site a “canopy” trap was operated, which was elevated to 6 m (2016) or 9 m (2017). Trap heights were changed between years in order to sample the midge community at multiple heights. Canopy traps were hoisted into tree canopies using a rope and pulley system and were placed in trees representing the dominant cover type in the stand (hardwood or pine). Out of the ten canopy traps on the property, six traps were located in stands composed primarily of hardwood tree species and four traps were located in majority pine stands (Fig. 1). Once trap sites were established, locations were not changed within years. Ropes were placed in trees using a “Chuckit!” brand tennis ball thrower (Doskocil Manufacturing Company, Arlington, TX, USA). Canopy trap ropes were attached to the tennis balls prior to using the tool to throw the tennis balls over tree branches. Ropes were subsequently dragged over the branch until the pulley was at the proper height and the rope was then secured to the tree trunk. Trapping was conducted one night per week for an 11-week period in 2016 (44 total trap nights per height) and a 12-week period in 2017 (120 total trap nights per height) during late spring and summer (June-August) each year. Data collected previously on this property indicated that midge abundances should be high during this trapping period.

**Fig. 2:**
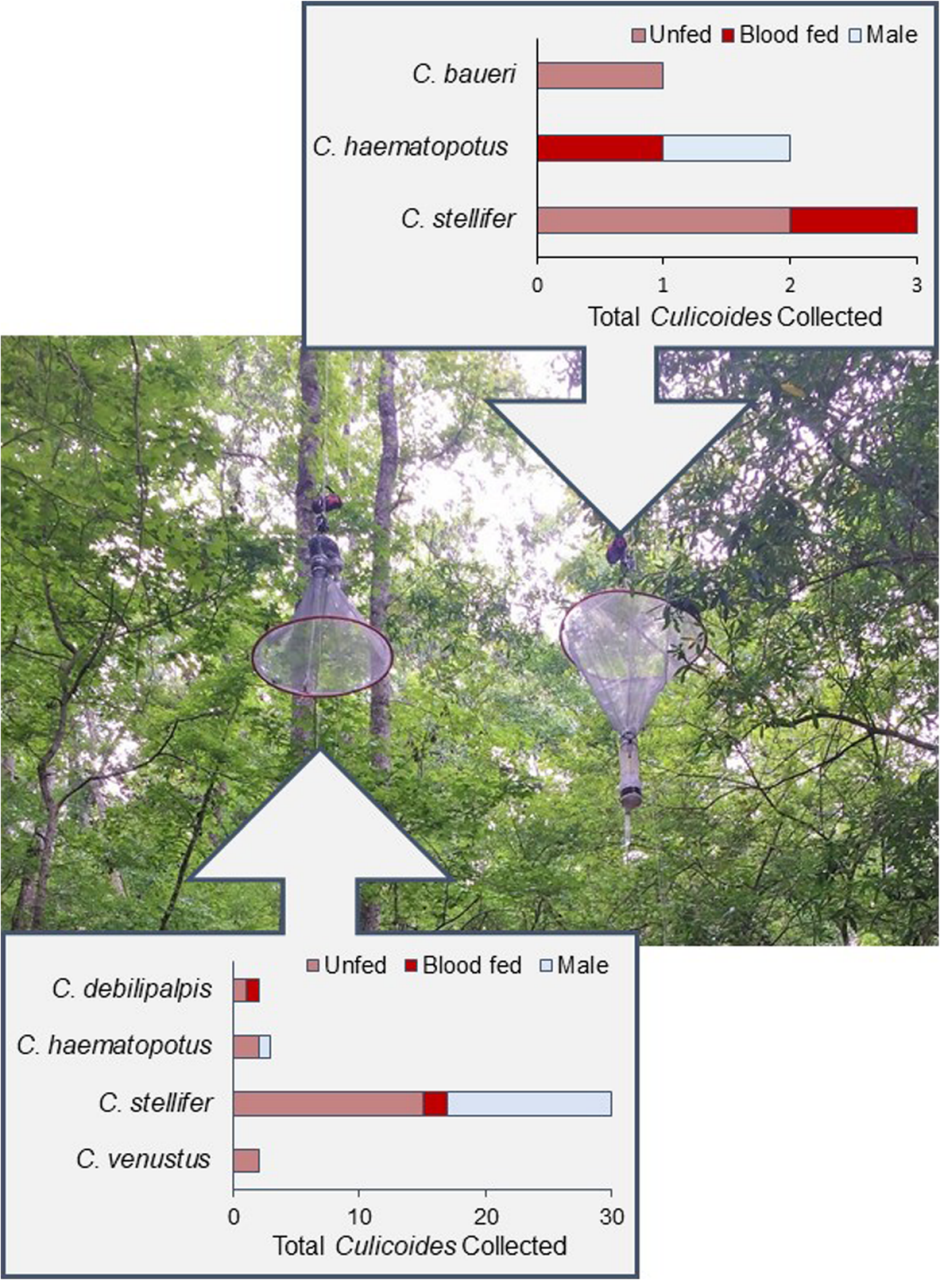
Funnel suction traps, biting midges captured and their physiological status. The trap at left collected insects as they ascended into the canopy and the trap at right collected insects as they descended from the canopy. Funnel traps were hung halfway between the canopy and ground traps (6 m) at two sites on the Gadsden county property for the duration of the study period in 2017

In addition to light traps, non-attractive “funnel suction traps” (Fig. 2) were used to determine the direction of travel (ascending or descending) of midges in the canopy. These traps also served as a control to determine whether the *Culicoides* collections in the forest canopy were solely due to the presence of an attractive light drawing midges upwards. Sampling with funnel suction traps was conducted during the summer of 2017. The traps were constructed of a large funnel of no-see-um netting that terminated at the intake of a suction trap (CDC miniature light trap with bulb removed). The funnel was 1 m in diameter at its opening and constructed of plastic rings with no-see-um netting (largest mesh opening 0.6 mm). The CDC trap (bulb removed) served to deposit insects into an attached 50mL conical tube containing 95% ethanol. At each site, one funnel suction trap was placed to collect insects as they descended from the canopy (Fig. 2, at right) and the other faced down to collect insects as they moved upwards (Fig. 2, at left). Traps were connected to 6V battery sources that were replaced every other day, allowing traps to run continuously throughout the week. Collection tubes were replaced weekly.

### Laboratory methods

*Culicoides* were sorted from bycatch and identified to species using morphological keys [17]. Physiological status of all midges was categorized as nulliparous, parous, gravid, blood-fed or male. Parous midges were recognized by the presence of accumulated red pigmentation on the abdominal cuticle [18]. Due to difficulty in differentiating nulliparous and parous *Culicoides venustus* Hoffman, these two physiological statuses were combined into a single group termed “unfed”.

Blood-meal analysis was conducted by polymerase chain reaction (PCR) to determine host use of blood-fed midges. DNA was extracted using Instagene Matrix (Bio-Rad Laboratories, Inc., Hercules, CA, USA) using the following extraction protocol. Each blood-fed individual was homogenized with 10 μl NaCl in a 1.5 ml microcentrifuge tube using a pestle. Room-temperature Instagene was added at 200 μl to each tube and vortexed. Tubes were incubated at 98 °C for 10 minutes and subsequently centrifuged at 3099× *g* for 5 min after which the supernatant was collected into a clean 1.5 ml tube.

Extracted DNA was then amplified by PCR using three primer sets and cycling conditions as described by Blosser et al. [19]. All samples were initially run using a mammalian and amphibian specific primer set targeting the *16S* region (forward: 5'-CTC CAT AGG GTC TTC TCG TCT T-3'; reverse: 5'-GCC TGT TTA CCA AAA ACA TCA C-3'). PCR protocol for this primer set was 94 °C for 2 min and 35 cycles of: 30 s at 94 °C, 30 s at 57 °C and 1 min at 72 °C. Samples were subsequently run using lizard specific *16S* region primers (forward: 5'-CTG ACC GTG CAA AGG TAG CGT AAT CAC T-3'; reverse: 5'-CTC CGG TCT GAA CTC AGA TCA CGT AGG-3') using the following protocol: 2 min at 94 °C and 35 cycles of 30 s at 94 °C, 30 s at 62.5 °C and 1 min at 72 °C. Finally, primers targeting the avian cytochrome *b* oxidase (forward: 5'-GGA CAA ATA TCA TTC TGA GG-3'; reverse: 5'-GGG TGG AAT GGG ATT TTG TC-3') were used with the following protocol: 2 min at 94 °C and 35 cycles of 30 s at 94 °C, 30 s at 60 °C and 1 min at 72 °C. All protocols included a negative control (molecular grade water) and a positive control.

Agarose gel electrophoresis was used to determine presence of PCR amplicons, using a 1% agarose gel (100 V for 35 min). Amplicons were sent to a commercial laboratory for Sanger sequencing (Eurofins Genomic, Louisville, Kentucky, USA). Sequences were then compared with available sequence information in the GenBank database (National Institutes of Health: National Center for Biotechnology Information) using BLAST (Basic Local Alignment Search Tool). Samples with identity matches of ≥ 95% and query coverages of 75% or higher were considered a host species match.

### Data analysis

All statistical analyses looking at individual species were restricted to those species with greater than 100 individuals collected per sample year. Individual species abundance was analyzed using negative binomial regression analysis to account for overdispersion of data and unequal mean and variance [20, 21]. Negative binomial regression was also used to investigate differences in species abundance between traps located in stands composed of primarily hardwood or pine trees during the 2017 sampling year. Pearson’s chi-square test of independence was used to analyze whether relative proportions of each physiological status for each species were dependent upon trap height. Blood-meal analysis results were also analyzed using Pearson’s chi-square analysis to test for independence of host class use (Mammalia and Aves) between the two trap heights (canopy and ground). All analyses were performed using R software (The R Foundation for Statistical Computing, version 3.3.2).

## Results

### Total collections

For the 2016 trapping season, four trap comparisons were run for 11 weeks resulting in 44 trap nights per height (or 88 trap nights total). This resulted in a total of 4769 midges collected on the big game preserve. Out of the total, 1996 (41.85%) were collected in ground traps and 2773 (58.15%) were collected in canopy traps. These collections comprised 14 *Culicoides* species (Table 1). We observed that most species (9/14) were collected in greater abundance in the forest canopy. The species for which at least 100 specimens were collected in 2016 included *Culicoides haematopotus* Malloch (*n* = 914), *Culicoides stellifer* Coquillett (*n* = 3363) and *C. venustus* (*n* = 418).

**Table 1:** Total *Culicoides* collected per species and height during the 2016 and 2017 sampling period. Total trap nights were 88 (44 per height) for 2016 and 240 (120 per height) for 2017. Species for which at least 10 individuals were collected are shown^a^

Species	2016		2017		Total
	Canopy	Ground	Canopy	Ground	
*C. arboricola*	11	1	202	15	229
*C. beckae*	0	1	43	0	44
*C. bickleyi*	0	0	23	4	27
*C. biguttatus*	1	2	118	9	130
*C. crepuscularis*	2	0	38	0	40
*C. debilipalpis*	31	12	689	55	787
*C. haematopotus*	404	510	1218	191	2323
*C. insignis*	0	0	122	20	142
*C. nanus*	1	1	27	0	29
*C. pallidicornis*	0	0	14	2	16
*C. spinosus*	1	1	3	7	12
*C. stellifer*	2013	1350	20,231	1752	25,346
*C. venustus*	300	118	1976	140	2534
Other spp.	9	0	18	11	38
Total	2773	1996	24,722	2206	31,697

^a^Other species excluded from the table include *C. baueri* (*n* = 4), *C. furens* (*n* = 2), *C. guttipennis* (*n* = 9), *C. hinmani* (*n* = 6), *C. loisae* (*n* = 2), *C. ousairani* (*n* = 4), *C. paraensis* (*n* = 2), *C. scanloni* (*n* = 1), *C. torreyae* (*n* = 4), and *C. villosipennis* (*n* = 4)

In 2017, the trapping effort was increased to ten sites run for 12 consecutive weeks resulting in 120 trap nights per height (240 trap nights total). This effort collected 26,928 individuals from 22 *Culicoides* species. The distribution between trap heights was 2206 (8.19%) in ground traps and 24,722 (91.81%) in canopy traps. A majority of species collected during this year were found in the forest canopy (17/22 species; Table 1). The species for which greater than 100 midges were collected included *Culicoides arboricola* Root (*n* = 217), *Culicoides biguttatus* Coquillett (*n* = 127), *Culicoides debilipalpis* Lutz (*n* = 744), *C. haematopotus* (*n* = 1409), *Culicoides insignis* Lutz (*n* = 142), *C. stellifer* (*n* = 21,983) and *C. venustus* (*n* = 2116)*.*

### Ground versus canopy use by abundant species

Species for which at least 100 specimens were collected were analyzed to look for intraspecific height preferences. In 2016, none of the most abundant species showed a significant difference between canopy and ground traps (*C. haematopotus*: *df* = 45, *P* = 0.673; *C. stellifer*: *df* = 83, *P* = 0.249; *C. venustus*: *df* = 59, *P* = 0.052). In 2017, the species with significantly greater abundance in canopy collections included *C. arboricola* (*df* = 96, *P <* 0.001), *C. biguttatus* (*df* = 42, *P* < 0.001), *C. debilipalpis* (*df* = 156, *P <* 0.001), *C. haematopotus* (*df* = 192, *P* < 0.001,), *C. insignis* (*df* = 42, *P* < 0.001) and *C. stellifer* (*df* = 226, *P* < 0.001). There was no significant difference between ground and canopy collections for *C. venustus* (*df* = 58, *P* = 0.052) in 2017. No *Culicoides* spp. were captured in significantly greater numbers in ground traps.

### Funnel suction traps

Funnel suction traps were operated throughout the summer of 2017 and collected 43 biting midges. Out of 43 individuals, 37 (86%) were collected ascending into the forest canopy while six (13.95%) were collected descending towards the ground (Fig. 2). Five blood-engorged *Culicoides* were collected, four of which were successfully identified including two descending from the forest canopy: one *C. haematopotus* with a northern cardinal (*Cardinalis cardinalis*) blood meal and one *C. stellifer* with a white-tailed deer blood meal. The other two blood-engorged midges, one *C. debilipalpis* and one *C. stellifer*, were ascending into the forest canopy with white-tailed deer blood meals. The blood meal from one blood-engorged *C. stellifer* was not identified. The majority of midges collected moving into the forest canopy (52.5%) were nulliparous females.

### Habitat associations of abundant species

**Fig. 3:**
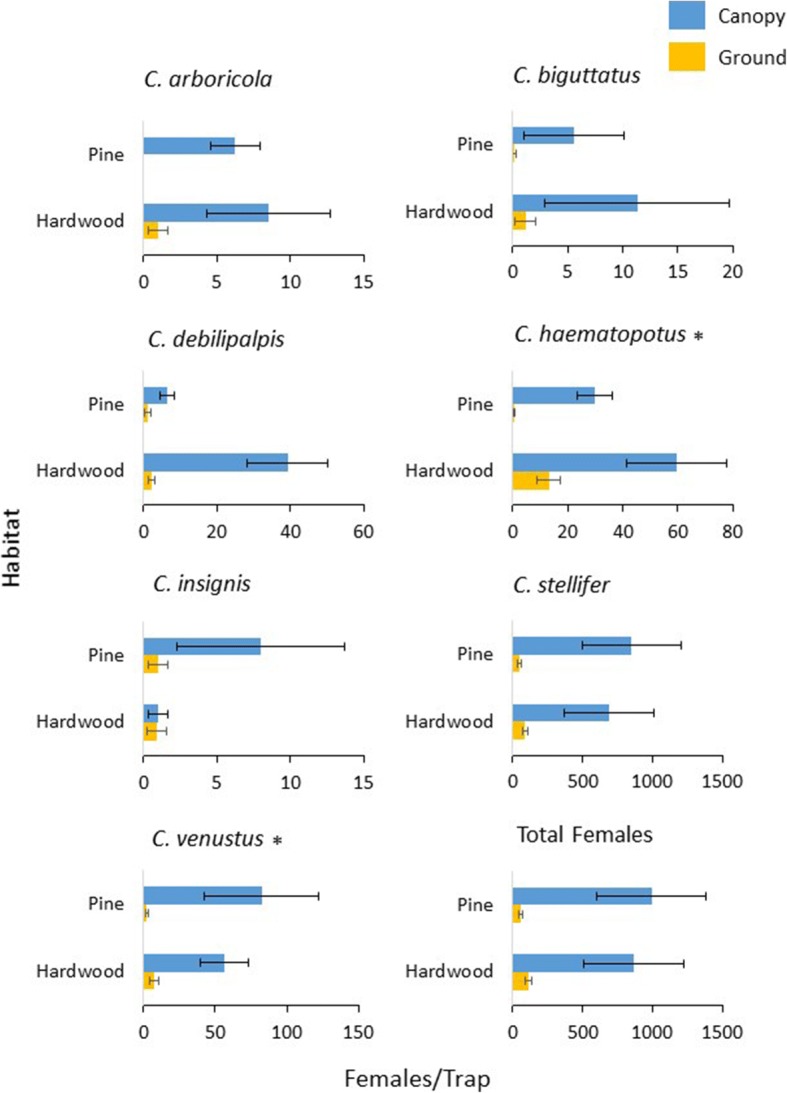
Habitat associations and vertical stratification of *Culicoides* species. Bars represent average females collected by CDC miniature light traps for which greater than 100 total individuals were collected. Asterisks denote a significant interaction between habitat and trap height at alpha = 0.05 (negative binomial regression) in 2017

Total abundance per habitat type for the seven abundant species was 12,725 in hardwood stands and 14,013 in pine stands. Binomial regression analysis indicated that the interaction of trap height and habitat type was not significant for *C. arboricola* (*df* = 47, *P =* 0.996), *C. biguttatus* (*df* = 27, *P =* 0.403), *C. debilipalpis* (*df* = 47, *P =* 0.076), *C. insignis* (*df* = 35, *P* = 0.181), and *C*. *stellifer* (*df* = 47, *P =* 0.637). A significant interaction of trap height and habitat type was identified for *C. haematopotus* (*df* = 47, *P <* 0.001) and *C. venustus* (*df* = 47, *P =* 0.042) (Fig. 3).

### Physiological status

**Fig. 4:**
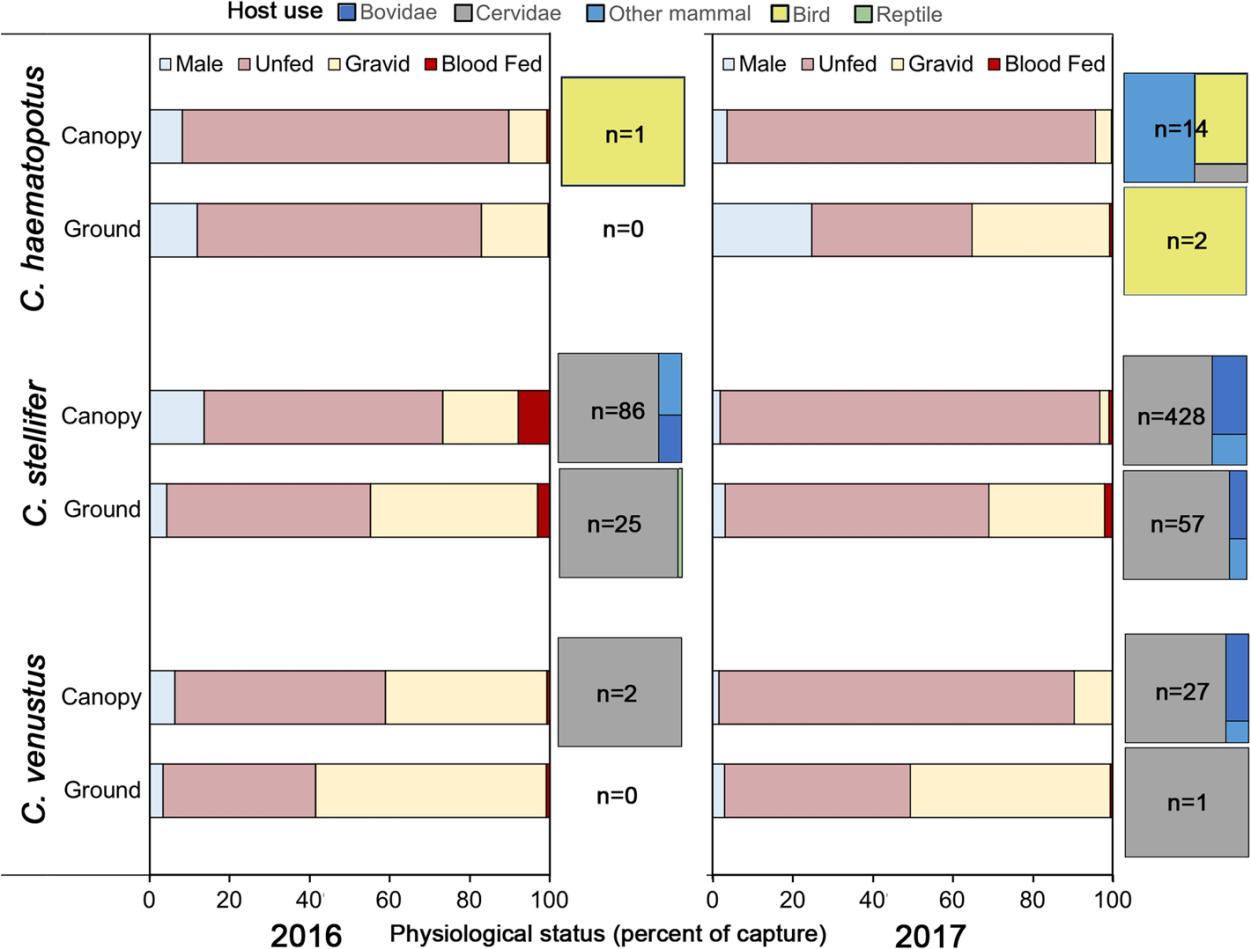
Vertical distribution, physiological status and host use of *Culicoides.* Parous and nulliparous groups were combined to account for difficulties in identifying parity in *C. venustus*. Treemaps represent the results of blood-meal analysis for that species in the ground and canopy traps each year. Only the three most commonly collected species are represented. The proportions of physiological statuses were significantly different in canopy versus ground traps for all species shown at *P =* 0.05. Private big game preserve, Gadsden Co. FL, 2016 and 2017

During 2016, there were significant differences between proportions of physiological statuses for all three species analyzed: *C. haematopotus*, *C. stellifer* and *C. venustus*. In 2017, significance was found for *C. arboricola*, *C. haematopotus*, *C. insignis*, *C. stellifer* and *C. venustus.* Physiological status proportions for *C. biguttatus* and *C. debilipalpis* were not significant and therefore independent of trap height. Chi-square results are reported in Table 2 and physiological status data for three of the most abundant species, *C. haematopotus*, *C. stellifer* and *C. venustus*, are shown in Fig. 4. In almost all cases, greater abundance of each physiological status was collected in canopy traps. Within trap heights, however, variable distributions of each physiological status were calculated (see Additional file 1: Table S1). Relative proportion of gravid females was higher in ground traps than canopy traps for all species with significant chi-square results for both study years. The relative proportions of all nulliparous females, with the exception of *C. haematopotus* in 2016, as well as unfed *C. venustus* were greater in canopy traps than ground traps for both years.

**Table 2:** Pearson’s chi-square results for distributions of physiological status in ground and canopy traps. Physiological statuses included were nulliparous, parous, gravid, blood-fed, and male. Nulliparous and parous counts were combined for *C. venustus* due to an inability to reliably determine parity. Males were excluded from the chi-square analysis for *C. biguttatus* since no male individuals were collected at either height. Private hunting preserve, Gadsden Co. FL, USA

Year	Species	*χ* ^2^	*df*	*P*-value
2016	*C. haematopotus*	26.991	4	<0.001^***^
	*C. stellifer*	266.05	4	<0.001^***^
	*C. venustus*	10.656	3	0.014^**^
2017	*C. arboricola*	81.905	4	<0.001^***^
	*C. biguttatus*	3.396	3	0.335
	*C. debilipalpis*	4.660	4	0.324
	*C. haematopotus*	81.905	4	<0.001^***^
	*C. insignis*	10.05	4	0.04^*^
	*C. stellifer*	107.83	4	<0.001^***^
	*C. venustus*	13.273	3	0.004^**^

^*^P < 0.05, ^**^P < 0.01, ^***^P < 0.001

### Blood meal analysis

During the 2016 sampling season, 222 total blood-engorged midges were collected, with 119 (53.6%) yielding identity matches of 95% or greater. Canopy and ground collections accounted for 94 (79%) and 25 (21%) specimens, respectively. Two avian blood meals were recorded from canopy level traps, one by *C. arboricola* on a black vulture (*Cathartes aura*) and the other by *C. haematopotus* on a red-eyed vireo (*Vireo olivaceus*). There were no avian blood meals taken by ground collected midges. The great majority of mammalian blood meals were from midges collected from the canopy traps (*n* = 92, 79.3% of total), while only 20.7% of mammalian blood meals were taken from ground collected midges (Fig. 4). Out of the 92 mammal blood meals taken from canopy midges, 82 were from large ground-dwelling ungulate species and nine were from humans. In addition, a blood meal was taken by *Culicoides crepuscularis* Malloch from a gray squirrel (*Sciurus carolinensis*), that could have been present in the canopy at the time of blood-feeding. Chi-square analysis indicated that the proportion of blood meals collected from each host class (Mammalia and Aves) was independent of trap height during the 2016 season (*χ*^2^ < 0.001, *df* = 1, *P =* 0.577).

A total of 790 blood-engorged female *Culicoides* were collected during the 2017 season, with 693 of these (87.7%) successfully matched to a host at the 95% identity match level. One species, *C. stellifer*, constituted 70% (*n* = 482) of the total blood meals in the dataset. The second greatest number of blood meals analyzed came from *C. debilipalpis* (20% of blood meals; *n* = 140). All other *Culicoides* species for which blood meals were analyzed constituted less than 5% of the total dataset. Canopy traps accounted for the vast majority of collections (89.2%, *n* = 618 blood meals), while ground traps only collected 75 blood-engorged females (10.8%). A total of 611 mammalian blood meals and seven avian blood meals were recovered from canopy collected midges while 73 mammalian and 2 avian blood meals were recovered from ground collected midges (Fig. 4). Mammalian blood meals from canopy collected midges were mostly (98.7%) from large ground-dwelling mammals except for one *C. stellifer* blood meal from a raccoon (*Procyon lotor*), one blood meal by *C. debilipalpis* on a *Peromyscus* sp*.* mouse, and six blood meals from eastern gray squirrels (four by *C. debilipalpis*, one by *Culicoides paraensis* Goeldi and one by *C. stellifer*). Chi-square analysis looking at trap height and host class indicated that host class proportions were independent of trap height for this analysis (*χ*^2^ = 0.342, *df* = 1, *P* = 0.268).

## Discussion

The results of this study indicate that adult *Culicoides* readily move into and exploit the forest canopy during each physiological state. Through the use of light baited traps, we identified that most *Culicoides* were collected in the forest canopy both years, although a much greater proportion of midges were collected in the canopy during the 2017 sampling year. The relative dissonance observed between study years may be associated with the difference in total trap nights between years (44 per height in 2016 compared with 120 per height in 2017). This pattern could also be associated with the height at which canopy traps were placed between years. In 2016, traps were located at 6 m height, much lower than traps in 2017 that were placed at 9 m. Research has indicated that in hardwood stands, leaf area index (LAI) is generally highest in the upper third of the forest canopy [22]. This could have led to increased cover for insects higher into the forest canopy.

Our analysis identified many species found in significantly greater abundance in the forest canopy. While the impetus for this trend is not fully understood, this finding has implications for the collection and quantification of local *Culicoides* populations of interest. Two of these species, *C. debilipalpis* and *C. stellifer*, are both suspected vectors for EHDV [23], and *C. insignis* is a confirmed BTV vector [24]. Quantifying abundance of these species is an important aspect of understanding the epidemiology of these veterinary pathogens. Many ongoing studies investigating *Culicoides* of veterinary importance place traps at low heights or fail to mention trap height placement [25, 26]. The data collected during the present study indicate that this practice may lead to biased sampling and inaccurate approximations of vector abundance.

While the majority of midges in both years were collected in the forest canopy, exceptions did occur and included species such as *Culicoides beckae* Wirth & Blanton and *C. biguttatus* in 2016 and *Culicoides loisae* Jamnback, *Culicoides scanloni* Wirth & Hubert, and *Culicoides torreyae* Wirth & Blanton in 2017*.* Very few specimens were caught for these species (10 total individuals), which prevented meaningful statistical analysis. As of now, it is unclear whether these species are selecting lower vertical strata or if this observation is a by-product of the low sample size collected from these species. Additional studies are warranted to better understand the ecology of rare *Culicoides* species. It is important to note that two of these species, *C. loisae* and *C. torreyae*, are not strongly hematophagous and likely do not rely solely on blood meals for egg production [17]. For this reason, it is unlikely that low flight habits are associated with searching for host animals.

While other studies investigating canopy use by *Culicoides* spp. and other hematophagous Diptera have used light- or CO_2_-baited traps [9, 11, 15], there are concerns that such collections are biased by the presence of baits. For this reason, the present study supplemented light trap collections with unbaited funnel suction traps, which successfully collected midges as they moved between the canopy and ground level without the use of an attractive bait. Further, our funnel traps only covered roughly 4 m^2^ of the property’s total area (200 ha). If our findings are extrapolated over the full area of the preserve, this indicates a substantial movement of *Culicoides* (2.1 million individuals) through vertical space during the study period. It is important to note, however, that while funnel traps collected various physiological statuses from a variety of species, no gravid individuals were collected using this method. It is unclear whether this is due to relatively low sample sizes collected with this method or if gravid individuals are not transitioning between strata as often as light traps indicated. Furthermore, it is possible the fine mesh used for collecting *Culicoides* in funnel suction traps were viewed by the midges as obstacles or enclosures rather than open space for transition. The low sample sizes collected could be partially due to the midges actively avoiding entering this enclosed space.

Forest canopies dominated by hardwood versus pine trees are fundamentally different habitats due to differences in crown density, leaf area index, and retained moisture [27, 28]. For two species, *C. haematopotus* and *C. venustus*, a significant interaction between trap height and habitat type was found, which indicated that for these two species, habitat may be an important factor in how midges stratify vertically between the ground and canopy. Both *C. haematopotus* and *C. venustus* are ground-breeding species, and as such, it is unlikely that these habitat preferences are due to increased prevalence of tree-associated breeding habitats such as tree-holes [17]. Despite lower retained moisture and crown density associated with pine stands [28], a greater abundance of *Culicoides* was detected from pine habitats overall, although this result was not significant. It is possible that this result could be due to the lower leaf area index of pine stands [27] leading to better visibility of the black light in these canopies compared to hardwood canopies.

Height did play a role in the proportion of the different physiological statuses collected for certain *Culicoides* species, suggesting that midges were using vertical space differently based on their physiological status. In each species where chi-square results indicated significantly disproportionate physiological status values, gravid individuals comprised a greater proportion of the total abundance in ground traps than in canopy traps. Despite this, overall abundance of total midges and gravid midges was greater in the canopy compared with ground traps. This is in agreement with the results of Venter et al. [9] that found greater abundance of gravid *C. imicola* as trap height was increased from 0.6 m to 2.8 m, while the relative proportion of gravid individuals decreased as height increased. The greater proportion of gravid individuals collected in ground traps may be using lower strata more often to search for suitable oviposition sites. This would be in agreement with research on mosquitoes that found greater collections of gravid *Culex pipiens* in ground catch basin traps than in canopy traps, which the authors state is probably due to gravid females seeking out oviposition sites [29]. For three of the midge species, *C. haematopotus*, *C. stellifer* and *C. venustus*, greater proportions of gravid individuals in ground traps can be explained by their larval development sites, which are typically moist substrate along water bodies, muddy puddles and muddy hoofprints [17]. This does not account for those species which are known container (treehole) breeders, including *C. arboricola* and *C. debilipalpis*, indicating that other factors, such as body mass, may be contributing to the use of lower strata by gravid females. In addition to tree-holes, *C. arboricola* has been documented ovipositing in woody debris and tree stumps [17], so low availability of tree-holes could lead this species to oviposit in woody debris on or near the ground. Greater proportions of unfed *C. venustus* and nulliparous midges were often found in the forest canopy, although the reason for this pattern is more challenging to parse out. This trend could be related to increased sugar feeding options, locating more suitable conditions for survival, or blood meal availability.

One of the objectives of the present study was to determine whether midges collected in the forest canopy were collected at the height representative of their preferred host range. Our analyses indicated that there was no significant association between the height at which blood-engorged midges were collected and the class of vertebrate they had been feeding upon during either study year. These data do not agree with the hypothesis that collection height can be used to determine host preferences [15]. This hypothesis is further challenged by the presence of canopy-dwelling mammals such as eastern gray squirrels (*Sciurus carolinensis*) and southern flying squirrels (*Glaucomys volans*)*.* For these reasons, using trap height to make inferences on host breadth is likely not an effective method of inferring host range and alternative methods should be used when possible. The distribution, number, and type of sensory pits on the antennal flagellomeres and maxillary palps of *Culicoides* has been proposed as another method to determine host preference [30, 31]. This hypothesis was not tested in the present study due to low blood-fed *Culicoides* species diversity but is an important topic for future studies. While our data do not support the hypothesis that collection height determines host-preference, the large ruminant abundance on this property was artificially high compared with a more natural system due to stocking and exclusion of predators. It is possible that the great abundance of large ruminants and their volatiles could have attracted more midges down from the canopy than in a more natural system where large hoofstock is not as readily available.

Very few data on the distances moved by blood-engorged Diptera are available, however Edman & Bidlingmayer [32] demonstrated that blood-engorged females of nine mosquito species flew at least one mile after engorgement [32]. Data on horizontal flight distance by blood-engorged *Culicoides* spp. are not available and is an important topic for future research. However, our data indicate that blood-engorged *Culicoides* are feeding on the ground and moving vertically up to 9 m into the forest canopy to digest their blood-meals.

Our finding that midges occupy the forest canopy in great abundance also indicates that current calculations of vectorial capacity may be low for some vector *Culicoides* species if trap height is not being taken into account. Garrett-Jones [33] identified this potential pitfall as an area of concern in calculating vectorial capacity for malarial vectors: blood-engorged arthropods tend to move and are not always collected in the areas where they originally fed. Not only was a great abundance of midges collected in the forest canopy at heights that may not typically be investigated, but a majority of blood-engorged midges in the canopy had fed upon large ungulates, including those susceptible to pathogens such as EHDV and BTV. One component of the vectorial capacity equation is the biting rate of the vector on the affected host. If blood-meal analysis is being used to investigate biting rates for vectorial capacity calculations, biting rate could be vastly underestimated by only sampling midges at ground level and underestimating a large proportion of midges that feed on ungulates on the ground and then ascend into the canopy level.

While our data demonstrate that large numbers of *Culicoides* can be encountered in tree canopies, the reason for this vertical movement is unknown, but it appears that this movement is likely not associated with host use and blood-feeding behavior. Other potential explanations for this movement include locating an optimal microclimate for survival or finding floral and extra-floral nectaries in trees. The microclimate of the forest canopy is variable at different heights, with higher canopy receiving more radiation, light and heat, and experiencing greater diurnal fluctuations and lower canopy maintaining more humid, cooler conditions and more diurnal stability [28]. Future studies should investigate if microclimate explains vertical habitat selection by *Culicoides* species.

The presence of attractive sugar sources including floral and extra-floral nectaries could entice midges into the tree canopy. A study conducted by Kaufmann et al. [34] in Europe found that around 80% of *Culicoides* collected at a farm were fructose-positive indicating frequent sugar access across all physiological stages. This study also found an increase in longevity of midges with access to ample sugar in addition to blood meals; however, Kaufmann et al. [34] also found that increased longevity was not associated with extrafloral nectaries specifically. Mullens [35] found that sugar-feeding was uncommon in *C. sonorensis* (as *C. variipennis*), with only up to 24% sugar-feeding detected in parous individuals. *Culicoides* represent a diverse assemblage of species with different characteristics and complex behaviors, so we speculate that vertical stratification may be linked to multiple driving forces during each physiological status. Observations such as gravid midges present at ground traps or unfed midges in canopy traps are likely due to differences in immediate physiological needs between individuals’ physiological states.

While this research has added valuable information on important *Culicoides* species present in Florida, many questions remain unanswered. It is unclear how frequently midges travel between the ground and canopy and at what time of day these transits are most likely to occur. A 1955 study by Snow [36] identified diel movements of *C. haematopotus* and *Culicoides guttipennis* Coquillett*,* which were mainly in the forest canopy throughout the day and descended to ground level to feed during the evening. This would be an excellent future direction for research into this phenomenon. Future studies should also trap along forest edges in addition to within the forest to look for abundance differences between these two sites, which have different canopy characteristics [37]. This could have implications for the spread of *Culicoides-*borne diseases to farms that border forest edges. The biggest question that remains is the main imperative driving midges into the forest canopy and whether this imperative is maintained through different physiological statuses.

## Conclusions

The present study has demonstrated a great abundance of *Culicoides* biting midges present in forest canopies in Florida, USA. The abundance of *C. arboricola*, *C. biguttatus*, *C. debilipalpis*, *C. haematopotus*, *C. insignis* and *C. stellifer* was significantly higher in the canopy than in ground collections in one study year. Physiological status was significantly dependent on trap height for *C. haematopotus*, *C. stellifer* and *C. venustus* in 2016, and for *C. arboricola*, *C. haematopotus*, *C. insignis*, *C. stellifer* and *C. venustus* in 2017. Despite speculation that host breadth could be inferred through height at collection, 98.6% of total blood-engorged midges collected in the canopy were found to have fed upon ground-dwelling mammals. These data not only add to our knowledge on these understudied *Culicoides* species, but also provide valuable insight into opportunities for surveillance and control of biting midge species of veterinary importance in Florida.

## Additional file

Additional file 1: **Table S1.** Physiological status distribution for ground and canopy collected *Culicoides* in 2016 and 2017 with relative proportion in parentheses. Only species with > 100 individuals collected are shown. (DOCX 23 kb)

## References

[1] Ulyshen MD (2011). Arthropod vertical stratification in temperate deciduous forests: implications for conservation-oriented management. Forest Ecol Manag.

[2] Černý O, Votýpka J, Svobodova M (2011). Spatial feeding preferences of ornithophilic mosquitoes, blackflies and biting midges. Med Vet Entomol.

[3] Cortez AM, Silva VPM, Quieroz PVS, Andrade HTA, Loiola MIB, Ximenes MFFM (2007). Vertical stratification and development aspects of phlebotomine sand flies (Diptera: Psychodidae) in an area of Atlantic forest tree species in a metropolitan region in northeastern Brazil. J Vector Ecol.

[4] Maguire DY, Robert K, Brochu K, Larrivée M, Buddle CM, Wheeler TA (2014). Vertical stratification of beetles (Coleoptera) and flies (Diptera) in temperate forest canopies. Environ Entomol.

[5] Anderson JF, Andreadis TG, Main AJ, Kline DL (2004). Prevalence of West Nile virus in tree canopy-inhabiting *Culex pipiens* and associated mosquitoes. Am J Trop Med Hyg.

[6] Mellor PS, Boorman J, Baylis M (2000). *Culicoides* biting midges: their role as arbovirus vectors. Annu Rev Entomol.

[7] Romero-Alvarez D, Escobar LE (2017). Oropouche fever, an emergent disease from the Americas. Microbes Infect.

[8] Ben-Chetrit E, Schwartz E (2015). Vector-borne diseases in Haiti: a review. Travel Med Infect Di.

[9] Venter GJ, Hermanides KG, Boikanyo SNB, Majatladi DM, Morey L (2009). The effect of light trap height on the numbers of *Culicoides* midges collected under field conditions in South Africa. Vet Parasitol.

[10] Veras RS, Castellón EG (1998). *Culicoides* Latreille (Diptera: Certopogonidae) in Brazilian Amazon. V. Efficiency of traps and baits and vertical stratification in the forest reserve Adolpho Ducke. Rev Bras Zool.

[11] Henry LG, Adkins TR (1975). Vertical distribution of biting midges in coastal South Carolina. Ann Entomol Soc Am.

[12] Swanson DA, Adler PH, Malmqvist B (2012). Spatial stratification of host-seeking Diptera in boreal forests of northern Europe. Med Vet Entomol.

[13] Service MW (1971). Adult flight activities of some British *Culicoides* species. J Med Entomol.

[14] Bennett GF (1960). On some ornithophilic blood-sucking Diptera in Algonquin Park, Ontario, Canada. Can J Zool.

[15] Swanson DA, Adler PH (2010). Vertical distribution of haematophagous Diptera in temperate forest of the southeastern U.S.A. Med Vet Entomol.

[16] Tanner GD, Turner EC (1974). Vertical activities and host preferences of several *Culicoides* species in a southwestern Virginia forest. Mosq News.

[17] Blanton FS, Wirth WW (1979). The Sand Flies (*Culicoides*) of Florida (Diptera: Ceratopogonidae).

[18] Akey DH, Potter HW (1979). Pigmentation associated with oogenesis in the biting fly *Culicoides variipennis* (Diptera: Ceratopogonidae) determination of parity. J Med Entomol.

[19] Blosser EM, Stenn T, Acevedo C, Burkett-Cadena ND (2016). Host use and seasonality of *Culex* (*Melanoconion*) *iolambdis* (Diptera: Culicidae) from eastern Florida, USA. Acta Trop.

[20] Rigot T, Drubbel MV, Delécolle JC, Gilbert M (2013). Farms, pastures and woodlands: the fine-scale distribution of Palearctic *Culiocides* spp. biting midges along an agro-ecological gradient. Med Vet Entomol.

[21] Dinesh DS, Das P, Picado A, Davies C, Speybroeck N, Boelaert M, Coosemans M (2008). The efficacy of indoor CDC light traps for collecting sandfly *Phlebotomus argentipes*, vector of *Leishmania donovani*. Med Vet Entomol.

[22] Vose JM, Sullivan NH, Clinton BD, Bolstad PV (1995). Vertical leaf area distribution, light transmittance, and application of the Beer-Lambert Law in four mature hardwood stands in the southern Appalachians. Can J For Res.

[23] Mullen GR, Hayes ME, Nusbaum KE (1985). Potential vectors of bluetongue and epizootic hemorrhagic disease viruses of cattle and white-tailed deer in Alabama. Prog Clin Biol Res.

[24] Tanya VN, Greiner EC, Gibbs EPJ (1992). Evaluation of *Culicoides insignis* (Diptera: Ceratopogonidae) as a vector of bluetongue virus. Vet Microbiol.

[25] Aybar CAV, Juri MJD, Lizarralde de Grosso MS, Spinelli GR. Spatial and temporal distribution of *Culicoides insignis* and *Culicoides paraensis* in the subtropical mountain forest of Tucumán, northwestern Argentina. Fla Entomol. 2011;94:1018–25.

[26] Brugger K, Köfer J, Rubel F (2016). Outdoor and indoor monitoring of livestock-associated *Culicoides* spp. to assess vector-free periods and disease risks. BMC Vet Res.

[27] Iiames JS, Cooter E, Schwede D, Williams J. A comparison of simulated and field-derived leaf area index (LAI) and canopy height values from four forest complexes in the southeastern USA. Forests. 2017; 10.3390/f9010026. 10.3390/f9010026 PMC5954438 29780445

[28] Parker GG, Lowman MD, Nadkarni NM (1995). Structure and microclimate of forest canopies. Forest Canopies.

[29] Anderson JF, Andreadis TG, Main AJ, Ferrandino FJ, Vossbrinck CR (2006). West Nile virus from female and male mosquitoes (Diptera: Culicidae) in subterranean, ground, and canopy habitats in Connecticut. J Med Entomol.

[30] Braverman Y, Hulley PE (1979). The relationship between the numbers and distribution of some antennal and palpal sense organs and host preference in some *Culicoides* (Diptera: Ceratopogonidae) from southern Africa. J Med Entomol.

[31] Isberg E, Hillbur Y, Ignell R (2013). Comparative study of antennal and maxillary palp olfactory sensilla of female biting midges (Diptera: Ceratopogonidae: *Culicoides*) in the context of host preference and phylogeny. J Med Entomol.

[32] Edman JD, Bidlingmayer WL (1969). Flight capacity of blood-engorged mosquitoes. Mosq News.

[33] Garrett-Jones C (1964). The human blood index of malaria vectors in relation to epidemiological assessment. Bull World Health Organ.

[34] Kaufmann C, Mathis A, Vorburger C (2014). Sugar-feeding behavior and longevity of European *Culicoides* biting midges. Med Vet Entomol.

[35] Mullens BA (1985). Age-related adult activity and sugar feeding by *Culicoides variipennis* (Diptera: Ceratopogonidae) in southern California, USA. J Med Entomol.

[36] Snow WE (1955). Feeding activities of some blood-sucking Diptera with reference to vertical distribution in bottomland forest. Ann Entomol Soc Am.

[37] Sherich K, Pocewicz A, Morgan P (2007). Canopy characteristics and growth rates of ponderosa pine and Douglas-fir at long established forest edges. Can J For Res.

