# Supplementary material for: Vertical stratification of Culicoides biting midges at a Florida big game preserve
Source: Parasit Vectors. 2018 Sep 10;11:505. doi: 10.1186/s13071-018-3080-5 (PMC6131774; doi:10.1186/s13071-018-3080-5)
Supplement: Supplementary file 1 — Table S1. Physiological status distribution for ground and canopy collected Culicoides in 2016 and 2017 with relative proportion in parentheses. Only species with > 100 individuals collected are shown. (DOCX 23 kb) [file 13071_2018_3080_MOESM1_ESM.docx]

**Additional file 1. Table S1.** Physiological status distribution for ground and canopy collected *Culicoides* in 2016 and 2017 with relative proportion in parentheses. Only species with > 100 individuals collected are shown. Nulliparous and parous individuals of *C. venustus* were combined into a single group, unfed, due to difficulty seeing pigmentation on abdomen of parous females.

| **Year** | ***Culicoides* Species** | **Height** | **Nulliparous** | **Parous** | **Gravid** | **Blood fed** | **Male** | **Unfed (*venustus* only)** | **Total** |  |
| --- | --- | --- | --- | --- | --- | --- | --- | --- | --- | --- |
| 2016 | *haematopotus* | Canopy | 45 (0.111) | 285 (0.705) | 38 (0.094) | 3 (0.007) | 33 (0.082) | - | 404 |  |
|  |  | Ground | 85 (0.167) | 277 (0.543) | 85 (0.167) | 2 (0.004) | 61 (0.120) | - | 510 |  |
|  | *stellifer* | Canopy | 483 (0.240) | 717 (0.356) | 377 (0.187) | 158 (0.079) | 278 (0.138) | - | 2013 |  |
|  |  | Ground | 277 (0.205) | 411 (0.304) | 563 (0.417) | 41 (0.030) | 58 (0.043) | - | 1350 |  |
|  | *venustus* | Canopy | - | - | 121 (0.403) | 2 (0.007) | 19 (0.063) | 158 (0.527) | 300 |  |
|  |  | Ground | - | - | 68 (0.576) | 1 (0.009) | 4 (0.034) | 45 (0.381) | 118 |  |
| 2017 | *arboricola* | Canopy | 128 (0.634) | 38 (0.188) | 8 (0.040) | 3 (0.015) | 25 (0.124) | - | 202 |  |
|  |  | Ground | 2 (0.133) | 1 (0.067) | 1 (0.067) | 8 (0.533) | 3 (0.200) | - | 15 |  |
|  | *biguttatus* | Canopy | 18 (0.153) | 61 (0.517) | 37 (0.314) | 2 (0.017) | 0 | - | 118 |  |
|  |  | Ground | 1 (0.111) | 6 (0.667) | 2 (0.222) | 0 | 0 | - | 9 |  |
|  | *debilipalpis* | Canopy | 180 (0.261) | 132 (0.192) | 77 (0.112) | 159 (0.231) | 141 (0.205) | - | 689 |  |
|  |  | Ground | 11 (0.200) | 14 (0.255) | 7 (0.127) | 8 (0.145) | 15 (0.273) | - | 55 |  |
|  | *haematopotus* | Canopy | 540 (0.443) | 323 (0.265) | 183 (0.150) | 25 (0.021) | 147 (0.121) | - | 1218 |  |
|  |  | Ground | 44 (0.230) | 55 (0.288) | 64 (0.335) | 3 (0.016) | 25 (0.131) | - | 191 |  |
|  | *insignis* | Canopy | 41 (0.336) | 18 (0.148) | 11 (0.090) | 11 (0.090) | 41 (0.336) | - | 122 |  |
|  |  | Ground | 6 (0.300) | 6 (0.300) | 5 (0.250) | 0 | 3 (0.150) | - | 20 |  |
|  | *stellifer* | Canopy | 7790 (0.385) | 7218 (0.357) | 2953 (0.146) | 535 (0.026) | 1735 (0.086) | - | 20231 |  |
|  |  | Ground | 527 (0.301) | 755 (0.431) | 327 (0.187) | 62 (0.035) | 81 (0.046) | - | 1752 |  |
|  | *venustus* | Canopy | - | - | 516 (0.261) | 29 (0.015) | 307 (0.155) | 1124 (0.569) | 1976 |  |
|  |  | Ground | - | - | 56 (0.400) | 1 (0.007) | 20 (0.143) | 63 (0.450) | 140 |  |
